# Gender Diversity of Research Teams and Clinical Trial Enrollment

**DOI:** 10.1001/jamanetworkopen.2025.37667

**Published:** 2025-10-16

**Authors:** Harsh Gupta, Anupam B. Jena, Eric C. Sun

**Affiliations:** 1 Department of Economics, Stanford University, Stanford, California; 2 Department of Health Care Policy, Harvard Medical School, Boston, Massachusetts; 3 Department of Medicine, Massachusetts General Hospital, Boston, Massachusetts; 4 National Bureau of Economic Research, Cambridge, Massachusetts; 5 Department of Anesthesiology, Perioperative and Pain Medicine, Stanford University School of Medicine, Stanford, California

## Abstract

**Importance:**

Inequity in clinical trial enrollment, particularly the underenrollment of women, is an ongoing concern in biomedical sciences. The extent to which increasing gender diversity among research teams, particularly, study principal investigators, could address this inequity is unclear.

**Objective:**

To assess the association between principal investigator gender and the proportion of women participants in clinical trials.

**Design, Setting, and Participants:**

This cross-sectional analysis used clinical trials registered on ClinicalTrials.gov that started enrollment after January 1, 2007, and completed enrollment before September 1, 2021, to analyze how the proportion of women participants in clinical trials varied by principal investigator gender, accounting for the disease being studied and when a trial was conducted (analyses were performed in October 2024). In an analysis of mechanisms, this study assessed whether specific decisions by investigators that could influence enrollment of women (eg, selecting women as study staff or including pregnant patients) varied by principal investigator gender.

**Exposure:**

Principal investigator gender.

**Main Outcomes and Measures:**

Proportion of trial participants who are women.

**Results:**

Across 10 708 trials, 3151 (29.2%) had a woman principal investigator. After adjusting for potential confounders, mean enrollment of women in trials with a woman principal investigator was 54.1% (95% CI, 53.0%-55.1%) compared with 46.9% (95% CI, 46.3%-47.5%) for trials in which the principal investigator was a man (absolute adjusted difference, 7.3% [95% CI, 6.7%-7.9%]; *P* < .001). Secondary analyses found that trials with a woman principal investigator had a higher proportion of women staff and were less likely to exclude pregnant patients.

**Conclusions and Relevance:**

This cross-sectional study of a large sample of clinical trials found that those with a woman principal investigator enrolled a higher proportion of women participants, had a higher proportion of women study staff, and were less likely to exclude pregnant patients. These results suggest that inequity in clinical trial enrollment, particularly the underenrollment of women, is an ongoing concern in biomedical sciences.

## Key Points

**Question:**

Are clinical trials led by women principal investigators more likely to recruit women as clinical trial participants?

**Findings:**

In this cross-sectional analysis of 10 708 trials, 29.2% had a woman principal investigator. After adjusting for potential confounders, mean enrollment of women in trials with a woman principal investigator was 54.1% compared with 46.9% for trials in which the principal investigator was a man, a statistically significant difference.

**Meaning:**

These results suggest that inequity in clinical trial enrollment, particularly the underenrollment of women, is an ongoing concern in biomedical sciences.

## Teaser

This cross-sectional study explores whether greater representation of women in research teams is associated with increased trial enrollment of women participants.

## Introduction

Clinical trials are critical to biomedical innovation. Substantial resources are invested in trials—approximately 30 000 trials are conducted annually and the median cost per trial is approximately $35 million^1^—and the results of trials shape how care is provided to patients globally. Concerns exist, however, that enrollment of patients into clinical trials is often inequitable, with women and minoritized individuals being underrepresented; therefore, the generalizability of trial findings to these populations is uncertain.^2^ Although enrollment of women in clinical trials has increased over time,^3^ they remain underrepresented,^4^ particularly in areas such as cardiology,^5,6^ surgery,^7^ emergency medicine,^8^ and oncology.^9,10^ Several laws and regulations, such as the National Institutes of Health (NIH) Revitalization Act of 1993, have been promulgated to increase enrollment of women in clinical trials, which is important given that spending on medical research disparately favors diseases that affect men.^11^

Increasing enrollment of women in clinical trials could have important implications for population health. For instance, because there are sometimes large differences in drug responses between men and women,^12,13^ adequate enrollment of women is needed to assess these differences, which may guide treatment and dosing decisions. Indeed, as many as 10% to 20% of drugs newly approved by the US Food and Drug Administration (FDA) show differences in exposure and response by gender that necessitate population-specific prescribing.^14^ A crucial example of this issue is the case of zolpidem, which was approved in 1992 on the basis of a trial where women accounted for only 37% of trial participants^15^; nearly 20 years later the recommended dose for women was reduced by half on the basis of trials showing that women metabolize the drug more slowly. More than 700 traffic incidents were attributed to the inappropriate dosing.^16^

Underrepresentation of women in clinical trials may similarly impact how treatments are provided to women through residual exclusion, whereby physicians may be reluctant to prescribe medications to women if the underlying trials have mostly enrolled men.^17^ The National Academies recently noted that the lack of diversity in clinical trial enrollment imposes substantial economic costs, hinders the discovery of new therapies, and magnifies existing health care disparities.^18^ Many policies that existed in the past (and that continue to some extent to this day) have disincentivized the enrollment of women in clinical trials, such as regulations regarding the enrollment of pregnant people.^19,20^

Given the central role of clinical trials in generating scientific evidence to improve health, it is critical to understand what factors might leave women out of that process, as both participants in and leaders of clinical trials. One potential factor that may influence enrollment of women in clinical trials is whether trials themselves are led by women. Principal investigators set priorities for how trials are conducted, and an important, but unstudied, way to increase enrollment of women in clinical trials would be to ensure adequate representation of women among investigators. In theory, this could lead to greater investigator efforts to recruit and build trust among women trial participants. Outside medicine, women innovators are more likely to innovate in areas that serve women’s needs,^21,22^ and women authors are more attentive to sex and gender analyses in academic research.^23^ Within medicine, research has found improved clinical outcomes when women patients are cared for by women physicians.^24,25^

In this study, we assembled data on the gender composition of research study teams, including investigators and recruitment staff, and the gender composition of trial participants from more than 10 000 registered clinical trials. To understand whether greater representation of women in research teams could increase trial enrollment of women participants, we analyzed the association between the gender composition of study teams and the gender composition of enrolled patients.

## Methods

### Data Collection

Clinical trial data were obtained from the Aggregate Content of ClinicalTrials.gov dataset (AACT), which provides information on clinical trials registered on ClinicalTrials.gov, the largest clinical trial registry in the world, with more than 300 000 clinical trials currently registered. All trials conducted in the US and all trials relating to FDA approval are required to be registered in this database. AACT data included the names of principal investigator(s), number of men and women participants enrolled in the trial, Medical Subject Heading (MeSH) terms associated with the trial, year in which the trial started enrollment, and funding sources (eg, whether the trial received NIH funding). The study used publicly available data and was therefore exempted from human subject review and informed consent by Stanford University. The study followed the Strengthening the Reporting of Observational Studies in Epidemiology (STROBE) reporting guideline for cross-sectional studies.

### Study Population

Our sample consisted of all trials for adults in the AACT database that started enrollment after January 1, 2007, and completed enrollment before September 1, 2021. From this initial sample of 33 259 trials, we then applied the following exclusion criteria. First, we excluded trials that did not report any information about principal investigators (n = 20 262). Second, we excluded trials (n = 1308) with more than one principal investigator, although these excluded trials were used in a sensitivity analysis. Third, as described below, our primary independent variable was the inferred gender of the principal investigator, determined from a name-to-gender algorithm using the investigator’s first name. For our primary analysis, we excluded trials where the gender of the principal investigator could not be inferred with sufficient certainty based on first name; specifically, trials were excluded for which we were unable to identify the gender of the principal investigator with more than 75% certainty (n = 981), although we performed additional sensitivity analyses based on alternative definitions of this threshold and using several name-to-gender algorithms. After applying these criteria, our primary analysis sample included 10 708 trials (see eFigure 1 in Supplement a for a diagram outlining sample construction and eTable 1 in Supplement 1 for a comparison of included and excluded trials).

### Outcome and Exposure

The primary outcome was the proportion of women participants in a trial, which was calculated as the number of women participants divided by the total number of participants.

The independent variable of interest for our primary analysis was the inferred gender of a trial’s principal investigator. We imputed principal investigator gender based on first name as a probability, using a validated approach that uses publicly available data to estimate the proportion of female births with a given first name.^26^ We assigned principal investigator gender based on the gender package in R that uses large historical datasets to infer gender based on first names, including sources such as the US Social Security Administration, IPUMS USA, and the North Atlantic Population Project. In our baseline analysis, a principal investigator was considered to be a woman if the first name was associated with at least a 75% probability of being a woman and was considered to be a man if the first name was associated with at least a 75% probability of being a man, using the above name-to-gender algorithm.

As previously noted, studies whose principal investigator had a name that fell outside this threshold (ie, for whom gender could not be inferred with sufficient certainty) were excluded. However, we performed sensitivity analyses in which the threshold was raised to 90% for a given gender (a stricter threshold for identifying gender based on name) and a second analysis in which this threshold was lowered to 50% (a more lenient threshold). We also evaluated several alternative name-to-gender algorithms (eMethods in Supplement 1).

### Additional Covariates

We analyzed the association between principal investigator gender and the proportion of enrolled patients in a trial that were women, adjusting for covariates that might confound this association. First, we adjusted for the underlying disease(s) addressed by a given trial because diseases that primarily affect women may have a higher enrollment of patients who are women. If principal investigator gender is not randomly allocated across disease areas (eg, if diseases that more often affect women, such as breast cancer, have trials more often led by women), the failure to adjust for disease could bias our findings. To identify the disease associated with a trial, we used 3-digit *International Classification of Diseases, Ninth Revision (ICD-9)* codes.^27^ These *ICD-9* codes allowed us to granularly adjust for different diseases, such as breast cancer, prostate cancer, diabetes, and hypertension (*ICD-9* codes are not directly available on ClinicalTrials.gov). We obtained *ICD-9* codes by mapping the MeSH terms associated with a trial to *ICD-9* codes using the Unified Medical Language System from the National Library of Medicine (see eMethods in Supplement 1 for additional details). For certain descriptive analysis (Table), we aggregated the 3-digit *ICD-9* codes to coarser 1-digit *ICD-9* codes. These coarser codes were used only for descriptive analysis and not for the statistical analysis.

**Table.** Characteristics of the Study Population

Characteristic	Study participants, % (95% CI)		Difference, % (95% CI)	*P* value
	Men principal investigators (n = 7555)	Women principal investigators (n = 3153)		
No. of patients in trial, mean (SD)	423.0 (586.1)	715.3 (14 108.5)	292.5 (−85.8 to 670.7)	.50
No. of trial sites, mean (SD)	6.9 (30.0)	3.7 (17.9)	−3.1 (−4.3 to −2.0)	<.001
Phase 3 or 4	25.4 (24.4 to 26.3)	18.5 (17.0 to 20.0)	−6.8 (−8.6 to −5.1)	<.001
Disease categories				
Mental health	23.1 (20.1 to 25.4)	26.3 (22.8 to 29.8)	3.2 (−9.4 to 7.4)	.13
Circulatory	18.7 (16.7 to 20.1)	14.9 (11.9 to 18.1)	−3.7 (−7.5 to 0.0)	.050
Nervous system	18.6 (16.5 to 20.1)	20.0 (16.8 to 23.3)	1.5 (−2.4 to 5.3)	.50
Genitourinary	9.4 (7.8 to 11.0)	8.9 (6.5 to 11.3)	−0.5 (−3.3 to 2.3)	.70
Infection	4.5 (3.4 to 5.6)	3.5 (1.8 to 5.1)	−1.0 (−3.0 to 0.9)	.30
Enrollment of women	46.9 (46.3 to 47.5)	54.1 (53.1 to 55.0)	7.2 (6.1 to 8.4)	<.001

Second, because enrollment of women in trials has increased over time, a failure to account for this trend would lead to a spurious association between clinical trial leadership by women and enrollment of women as trial participants if the proportion of women principal investigators has also increased. To account for time trends in women investigators and trial participants, we adjusted for the year a study began enrollment (indicator variable). Third, we conducted sensitivity analyses using several additional covariates.

### Statistical Analysis

A multivariable linear regression was used to estimate the association between principal investigator gender and the share of women participants in a trial. The dependent variable was the percentage of women participants in a trial, and the independent variable of interest was an indicator variable for whether the trial principal investigator was a woman, as inferred based on first name. Additional covariates included indicator variables for disease category and year. This regression therefore estimated the absolute percentage change in women trial participants associated with a woman principal investigator, after adjusting for the disease being studied and the year a trial began. Sensitivity analyses included additional covariates in the regression analysis. Analyses were conducted in October 2024 using R, version 4.0.2 (R Foundation for Statistical Computing).

#### Sensitivity Analyses

We performed several sensitivity analyses. First, an integral part of our analysis was the identification of principal investigator gender based on first name. Using our primary name-to-gender algorithm, we conducted a sensitivity analysis in which the threshold for assigning gender was (1) increased from a 75% to 90% probability, a stricter threshold for accuracy, and (2) lowered to 50%, a less strict threshold. This analysis evaluated the sensitivity of our findings to alternative probabilistic thresholds of assigning principal investigator gender based on first name.

Second, our primary approach for assigning principal investigator gender was based on the gender package in R that uses large historical datasets to designate gender based on first names. A potential limitation of this algorithm, however, is its reliance on data sources from the US and North Atlantic countries, which may not apply to investigators with first names from other parts of the world. We therefore examined the sensitivity of our findings to 3 alternative name-to-gender algorithms (Gender API, genderize.io, and Namsor), which are also widely used in academia and industry^28,29^ and which use numerous data sources that cover multiple continents and countries. We began by examining the extent to which the gender inferred by our primary algorithm matched the gender inferred from these alternative algorithms. We then repeated our baseline analysis using inferred gender from each of these 3 algorithms. In addition, we conducted separate analyses for names of European and non-European origin to provide further validity to our use of these name-to-gender algorithms to reliably assess investigator gender (see eMethods in Supplement 1 for additional details).

Third, confounding from unobserved confounders is a potential source of bias in observational studies. We addressed this in 2 ways. First, we reestimated our models, including additional covariates such as trial phase (separate indicator variables for phases 1-3), whether the trial was funded by the NIH, trial size, and whether the trial was multicenter. We also considered interaction effects between these additional covariates and whether a trial had a woman principal investigator. We assessed the likelihood and impact of potential confounding by estimating the required strength of confounding that would be necessary to explain away our findings (see eMethods in Supplement 1 for additional details).^30^

Fourth, because our primary analysis excluded trials with more than one principal investigator, we performed an additional analysis in trials with more than one principal investigator to compare enrollment of women between trials with only men principal investigators, both men and women principal investigators, and only women principal investigators. We hypothesized a dose-response relationship (ie, that enrollment of women would be higher with more women principal investigators). We estimated a regression similar to our baseline analysis, except that our independent variable of interest was modified to consist of 2 variables: an indicator variable for whether all principal investigators in a trial were women and another for whether a trial had both men and women principal investigators. Fifth, our primary analysis adjusted for the disease being studied by a trial using fixed effects based on the 3-digit *ICD-9* code associated with the disease being studied. We considered alternative methods for adjusting for disease, such as adjusting for the NIH funding the trial and adjusting for 1-digit *ICD-9* codes. In addition, we also estimated a model in which we excluded diseases that solely affected men or women (eg, prostate cancer).

In addition to these analyses, we assessed possible mechanisms by which principal investigator gender could influence the gender composition of trial participants. First, studies suggest that racial and gender concordance can increase participation in medical services and clinical trials,^31,32,33^ especially because lack of trust is a barrier to clinical trial enrollment of women.^34,35,36,37,38^ We examined whether women principal investigators were more likely to use women site coordinators or investigators. We hypothesized that having more women site coordinators or facility investigators at trial sites could increase enrollment of women. For this analysis, we analyzed a different sample of the AACT data consisting of 24 009 trials that were actively recruiting as of September 1, 2021. Because these trials were actively recruiting (ie, had not closed enrollment), names of site coordinators or facility investigators at each site were still available online, allowing us to designate gender of these staff as well. This allowed us to examine the association between principal investigator gender and the gender composition of listed site coordinators or facility investigators among trials that were actively recruiting. We performed a linear regression in which the dependent variable was the proportion of a trial’s listed site coordinators or facility investigators that were women, and the key independent variable was an indicator for whether the trial principal investigator was a woman. Additional covariates included indicator variables for disease category and year. Notably, because ClinicalTrials.gov removes contact information of site coordinators or facility investigators once a trial has completed enrollment, and completion of enrollment was required for us to calculate the final gender composition of trial participants, we could not directly assess the impact of gender composition of site coordinators or facility investigators on gender composition of trial participants.

Second, due to historical restrictions,^19^ increased regulatory requirements,^20^ and perceived increased legal risk,^39^ many investigators exclude pregnant patients from clinical trials, even if doing so is not appropriate.^40^ We hypothesized that women principal investigators would be more willing to be as inclusive as possible of pregnant participants and would therefore be less likely to apply explicit exclusion criteria for pregnant patients. To test this hypothesis, we estimated a similar multivariable linear regression with the dependent variable being an indicator variable for whether the trial excluded pregnant patients, which we obtained from the AACT database. In addition, we also estimated a similar model that excluded trials that evaluated drugs that were known to be contraindicated in pregnant patients (Category X drugs).

## Results

Our study consisted of 10 708 trials, of which 3153 (29.4%) had a woman principal investigator. Trials led by women principal investigators were larger, were conducted at fewer trial sites, were less likely to involve phase 3 or phase 4 trials, reflected similar disease areas, and had a higher proportion of trial participants who were women (Table). Compared with trials excluded from our sample, trials in our sample involved fewer sites, had less total enrollment, were less likely to be late stage, and received less NIH funding (eTable 1 in Supplement 1). The proportion of trials led by women principal investigators and the share of trial participants who were women were stable throughout the study period (eFigure 2 in Supplement 1).

**Figure 1.**
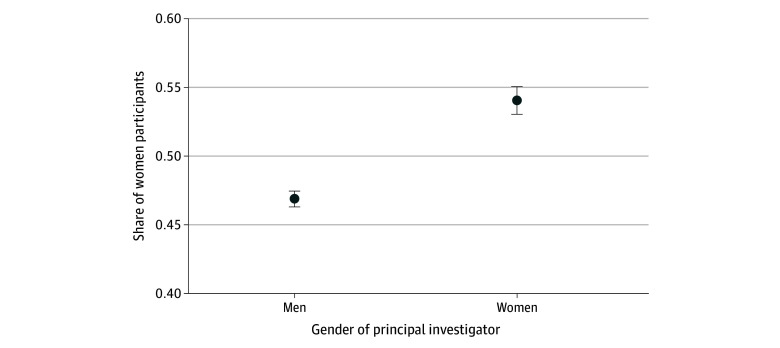
Enrollment of Women Participants in Clinical Trials Led by Men vs Women Principal Investigators Figure presents the adjusted share of participants who are women for trials led by men vs women principal investigators. Error bars indicate 95% CIs.

After multivariable adjustment, the adjusted proportion of women participants in trials was 54.1% (95% CI, 53.0%-55.1%) for trials with a woman principal investigator compared with 46.9% (95% CI, 46.3%-47.5%) for trials in which the principal investigator was a man (absolute adjusted difference, 7.3%; 95% CI, 6.7%-7.9%, *P* < .001) (Figure 1).

**Figure 2.**
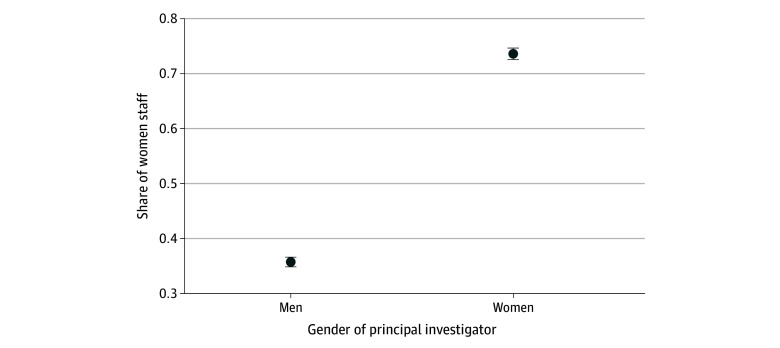
Gender Composition of Research Staff in Clinical Trials Led by Men vs Women Principal Investigators Figure presents the adjusted share of research staff who are women for trials led by men vs women principal investigators. Research staff include study site coordinators or site principal investigators. Error bars indicate 95% CIs.

Trials led by women principal investigators had a significantly higher share of site coordinators or investigators being women. For example, after multivariable adjustment, in trials with a woman principal investigator, 73.6% (95% CI, 72.5%-74.7%) of listed site coordinators or facility investigators were also women compared with 35.7% (95% CI, 34.8%-36.7%) of these staff for trials in which the principal investigator was a man (absolute adjusted difference, 39.3%; 95% CI, 37.5%-41.2%; *P* < .001) (Figure 2).

**Figure 3.**
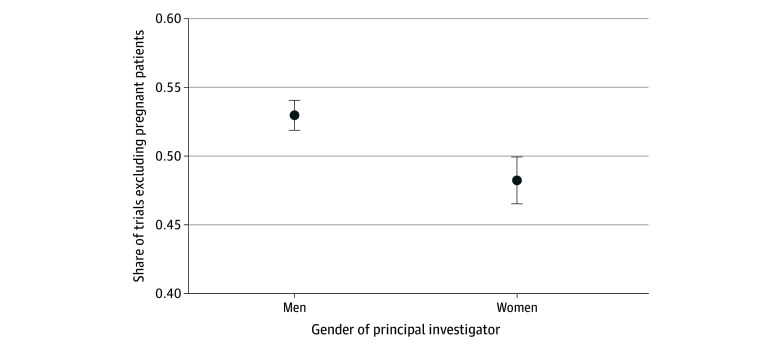
Exclusion of Pregnant Patients in Clinical Trials Led by Men vs Women Principal Investigators Figure presents the adjusted share of trials excluding pregnant patients for trials led by men vs women principal investigators. Error bars indicate 95% CIs.

Trials led by women principal investigators were also less likely to exclude pregnant patients. After multivariable adjustment, 48.2% (95% CI, 46.5%-50.0%) of trials with a woman principal investigator excluded pregnant patients compared with 53.0% (95% CI, 51.9%-54.1%) of trials led by a man (absolute adjusted difference, −4.8%; 95% CI, −6.0% to −3.7%; *P* < .001) (Figure 3).

Analyses of trials with more than one principal investigator found that enrollment of women increased with the involvement of women as principal investigators (eFigure 3 in Supplement 1), consistent with a dose-response relationship. Sensitivity analyses centered around alternative thresholds for identifying principal investigator gender produced similar results (eTable 2 in Supplement 1), as did analyses using alternative name-to-gender algorithms (eTables 3 and 4 in Supplement 1). Analyses suggested that confounding from unobservable factors would need to be implausibly large to explain away our findings (eTable 5 and eFigure 4 in Supplement 1) and sensitivity analyses that included additional potential confounders and different methods of adjusting for underlying disease in the regression produced similar results (eTables 6 and 7 in Supplement 1). We found no statistically significant interactions between a trial having a woman principal investigator and whether the trial was early vs late stage or was conducted in a single site vs multicenter (eTable 8 in Supplement 1). The association between women principal investigators and the inclusion of pregnant trial participants was unaffected by removing trials for drugs that are contraindicated among pregnant women (eFigure 5 in Supplement 1).

## Discussion

Although clinical trials are foundational to medicine, concerns exist that the enrollment of patients into trials is often inequitable, limiting trials’ applicability for underenrolled groups, such as women and racial and ethnic minority groups.^2^ The important implication of this inequity in trial representation is that treatments that are provided to women and minority populations today may lack the same level of evidence generated for other populations, posing issues of treatment safety and efficacy.

We hypothesized that the gender composition of a research team might influence the enrollment of women into clinical trials. We assembled a large database of clinical trials to examine whether having a woman principal investigator was associated with increased enrollment of women participants into trials. Our findings suggest that having a woman principal investigator is associated with a significant increase in enrollment of women into trials–an absolute increase of 7 percentage points or a 15% relative increase.

The underrepresentation of women as trial participants is both a symptom and a cause of broader health inequalities. Historically, spending on medical research has disproportionately favored diseases that affect men.^11^ Another bias, as shown in cardiovascular disease, has been the tendency to overlook differences in disease presentation and physiology between men and women, leading to sometimes missed or delayed diagnoses among women.^41^ In turn, the underrepresentation of women in clinical trials may increase disparities in population health between men and women through several avenues, including incorrect dosing for women,^12,13,15,16^ incorrect interpretation of trial results,^41^ or a reluctance by practitioners to prescribe treatments for women if a drug has not been sufficiently studied.^17^

In this light, our study highlights 2 important benefits of having more women principal investigators. First, increasing the relative number of women principal investigators may improve enrollment of women in clinical trials, thereby directly improving health by providing higher-quality evidence on safety and efficacy of medical treatments for women specifically. However, a second benefit with longer-term impact is that increasing the relative number of women principal investigators may also improve health by mitigating broader systemic biases, such as the failure to appreciate differences in disease presentation and pathophysiology between men and women^41^ or by reducing the use of disease models focused on men.^42^ Our findings are consistent with a larger literature suggesting that having more representative teams is associated with improved outcomes, such as previous work showing improved outcomes when women patients are cared for by women physicians.^24,25^ Although our analysis adjusted for disease area to better identify the potential effect of principal investigator gender on women’s participation in trials, the broader pattern of women principal investigators selecting into women’s health research represents an important complementary mechanism by which increased trial leadership by women could enhance overall trial participation among women.

Our study raises the question of why enrollment of women into trials may differ between men and women principal investigators. Implicit bias may play a role. In addition, existing policies place various incentives^43^ and disincentives^20^ for enrollment of women, and women principal investigators may weigh these incentives differently. For example, current policies impose additional regulatory requirements for enrolling pregnant patients.^20^ However, our study found that women principal investigators are less likely to exclude pregnant patients, suggesting that women principal investigators may be more willing to be as inclusive as possible in recruitment of women trial participants, even if doing so imposes additional regulatory requirements, cost, and effort.

Our study also potentially revealed another mechanism, which is that trials with a woman principal investigator were more likely to use women as site coordinators or facility investigators. Studies suggest that gender concordance is associated with improved outcomes^25,44^ and that it may improve participation in clinical trials.^31,32,33^ Women research staff may be more likely to recruit women participants or women participants may be more likely to enroll if the “face” of the trial to them is a woman, especially given studies suggesting that women clinicians tend to spend more time with their patients.^45^ We also consider this mechanism to be particularly important because it directly informs recent debates on the training and employment of the clinical and scientific workforce in health care.

In light of ongoing concerns regarding the underenrollment of women into clinical trials,^2^ our findings suggest that there may be societal benefits from policies aimed at increasing the number of trials with women principal investigators. Moreover, these findings raise important hypotheses and suggest that trials led by principal investigators who are either men or women may also increase enrollment of women by (1) more carefully considering whether pregnant women should be excluded from trials and (2) increasing women on the research team. Policies aimed at increasing incentives for women’s enrollment may also be helpful. For example, although there are appropriate concerns regarding the inclusion of pregnant people in trials, institutional review boards and funding agencies should carefully consider when the exclusion of pregnant people is needed. In addition, although many funding agencies have policies to promote the inclusion of women in clinical trials,^46^ reviewing agencies (eg, FDA) should likewise develop policies aimed at increasing enrollment of women.

### Limitations

Our study had several limitations. First, the study was limited to trials reported on ClinicalTrials.gov. Not all trials are required to report data to the website, and even among trials that are required to do so, compliance is imperfect.^47^ Trials in our sample appear to be smaller than the average trial, and our results should be interpreted with this fact in mind. Second, although we adjusted for several observable factors that could influence enrollment of women, confounding from unobservable factors is possible, although our analysis suggests such confounding would need to be implausibly large to explain away our findings. Third, our measurement of a study’s research staff was limited to the site coordinator or investigator and did not include other staff crucial to trial enrollment (eg, research coordinators). Fourth, our study used an investigator’s name to infer gender, which may not reflect the actual gender. Moreover, gender was inferred based on first name rather than self-report; the inference of gender was, however, highly stable across different name-to-gender algorithms and analyses. Fifth, although our findings suggest some mechanisms by which women principal investigators may increase enrollment of women participants, other mechanisms are possible. Qualitative research would be helpful.

## Conclusions

In this cross-sectional study of a large sample of clinical trials, we found that trials with a woman principal investigator had significantly greater enrollment of women trial participants, and we identified several mechanisms that may explain this association. These findings suggest that one way to improve patient representation in clinical trials and, importantly, to improve the foundation of evidence on which medical decisions are made for diverse populations would be to increase the diversity of clinical trial investigators. Inequity in clinical trial enrollment of women is a critical concern in medicine, in part because medical care provided to women today may lack the same level of evidence generated for other populations.

## Notes

Supplement 1. **eFigure 1**. Sample Construction **eFigure 2. **Time trends in women Principal Investigators and women participants **eFigure 3. **Enrollment of women participants in clinical trials according to gender composition of investigators in multi-Principal Investigator trials **eFigure 4. **Estimates of residual confounding bounds for select factors **eFigure 5.** Share of trials led by men versus women Principal Investigators that excluded pregnant patients, for drugs that are not contraindicated for pregnant women **eTable 1**. Comparison between included and excluded trials **eTable 2**. Findings using alternative thresholds for determining Principal Investigator gender **eTable 3. **Comparison of gender predictions using alternative algorithms **eTable 4. **Regression results using alternative algorithms to predict gender **eTable 5. **Estimate of residual confounding bounds **eTable 6. **Adjusted estimates with additional covariates **eTable 7**. Findings using additional methods of adjusting for disease **eTable 8**. Interactions with trial characteristics **eMethods**. Supplemental Methods
eReferences

Supplement 2.
Data Sharing Statement

## References

[1] Moore TJ, Zhang H, Anderson G, Alexander GC. Estimated costs of pivotal trials for novel therapeutic agents approved by the US Food and Drug Administration, 2015-2016. JAMA Intern Med. 2018;178(11):1451-1457. doi:10.1001/jamainternmed.2018.3931 30264133 PMC6248200

[2] Schwartz AL, Alsan M, Morris AA, Halpern SD. Why diverse clinical trial participation matters. N Engl J Med. 2023;388(14):1252-1254. doi:10.1056/NEJMp2215609 37017480

[3] Feldman S, Ammar W, Lo K, Trepman E, van Zuylen M, Etzioni O. Quantifying sex bias in clinical studies at scale with automated data extraction. JAMA Netw Open. 2019;2(7):e196700. doi:10.1001/jamanetworkopen.2019.6700 31268541 PMC6613296

[4] Ramasubbu K, Gurm H, Litaker D. Gender bias in clinical trials: do double standards still apply? J Womens Health Gend Based Med. 2001;10(8):757-764. doi:10.1089/15246090152636514 11703888

[5] Geller SE, Koch A, Pellettieri B, Carnes M. Inclusion, analysis, and reporting of sex and race/ethnicity in clinical trials: have we made progress? J Womens Health (Larchmt). 2011;20(3):315-320. doi:10.1089/jwh.2010.2469 21351877 PMC3058895

[6] Harris DJ, Douglas PS. Enrollment of women in cardiovascular clinical trials funded by the National Heart, Lung, and Blood Institute. N Engl J Med. 2000;343(7):475-480. doi:10.1056/NEJM200008173430706 10944565

[7] Hoel AW, Kayssi A, Brahmanandam S, Belkin M, Conte MS, Nguyen LL. Under-representation of women and ethnic minorities in vascular surgery randomized controlled trials. J Vasc Surg. 2009;50(2):349-354. doi:10.1016/j.jvs.2009.01.012 19631869 PMC2759770

[8] Safdar B, McGregor AJ, McKee SA, . Inclusion of gender in emergency medicine research. Acad Emerg Med. 2011;18(2):e1-e4. doi:10.1111/j.1553-2712.2010.00978.x 21314767

[9] Murthy VH, Krumholz HM, Gross CP. Participation in cancer clinical trials: race-, sex-, and age-based disparities. JAMA. 2004;291(22):2720-2726. doi:10.1001/jama.291.22.2720 15187053

[10] Baiu I, Titan AL, Martin LW, Wolf A, Backhus L. The role of gender in non-small cell lung cancer: a narrative review. J Thorac Dis. 2021;13(6):3816-3826. doi:10.21037/jtd-20-3128 34277072 PMC8264700

[11] Mirin AA. Gender disparity in the funding of diseases by the U.S. National Institutes of Health. J Womens Health (Larchmt). 2021;30(7):956-963. doi:10.1089/jwh.2020.8682 33232627 PMC8290307

[12] Yu Y, Chen J, Li D, Wang L, Wang W, Liu H. Systematic analysis of adverse event reports for sex differences in adverse drug events. Sci Rep. 2016;6:24955. doi:10.1038/srep24955 27102014 PMC4840306

[13] Zucker I, Prendergast BJ. Sex differences in pharmacokinetics predict adverse drug reactions in women. Biol Sex Differ. 2020;11(1):32. doi:10.1186/s13293-020-00308-5 32503637 PMC7275616

[14] Ramamoorthy A, Pacanowski MA, Bull J, Zhang L. Racial/ethnic differences in drug disposition and response: review of recently approved drugs. Clin Pharmacol Ther. 2015;97(3):263-273. doi:10.1002/cpt.61 25669658

[15] Statistical Review and Evaluation. Accessed December 18, 2023. https://www.accessdata.fda.gov/drugsatfda_docs/nda/pre96/019908_S000_STATR.pdf

[16] Questions and Answers: Risk of Next-Morning Impairment After Use of Insomnia Drugs; FDA Requires Lower Recommended Doses for Certain Drugs Containing Zolpidem (Ambien, Ambien CR, Edluar, and Zolpimist). 2018. Accessed December 18, 2023. https://www.fda.gov/drugs/drug-safety-and-availability/questions-and-answers-risk-next-morning-impairment-after-use-insomnia-drugs-fda-requires-lower

[17] Minkoff H, Moreno JD, Powderly KR. Fetal protection and women’s access to clinical trials. J Womens Health. 1992;1(2):137-140. doi:10.1089/jwh.1992.1.137 11654036

[18] Bibbins-Domingo K, Helman A, eds. Improving Representation in Clinical Trials and Research: Building Research Equity for Women and Underrepresented Groups. National Academies of Sciences, Engineering, and Medicine; 2022:1-3. 36137057

[19] Kons KM, Wood ML, Peck LC, . Exclusion of reproductive-aged women in COVID-19 vaccination and clinical trials. Womens Health Issues. 2022;32(6):557-563. doi:10.1016/j.whi.2022.06.004 36075817 PMC9197956

[20] Frew PM, Saint-Victor DS, Isaacs MB, . Recruitment and retention of pregnant women into clinical research trials: an overview of challenges, facilitators, and best practices. Clin Infect Dis. 2014;59(suppl 7):S400-S407. doi:10.1093/cid/ciu726 25425718 PMC4303058

[21] Koning R, Samila S, Ferguson JP. Who do we invent for? patents by women focus more on women’s health, but few women get to invent. Science. 2021;372(6548):1345-1348. doi:10.1126/science.aba6990 34140388

[22] Koning R, Samilia S, Ferguson JP. Inventor gender and the direction of invention. AEA Pap Proc. 2020;110:250-254. doi:10.1257/pandp.20201045

[23] Nielsen MW, Andersen JP, Schiebinger L, Schneider JW. One and a half million medical papers reveal a link between author gender and attention to gender and sex analysis. Nat Hum Behav. 2017;1(11):791-796. doi:10.1038/s41562-017-0235-x 31024130

[24] Wallis CJD, Jerath A, Coburn N, . Association of surgeon-patient sex concordance with postoperative outcomes. JAMA Surg. 2022;157(2):146-156. doi:10.1001/jamasurg.2021.6339 34878511 PMC8655669

[25] Miyawaki A, Jena AB, Rotenstein LS, Tsugawa Y. Comparison of hospital mortality and readmission rates by physician and patient sex. Ann Intern Med. 2024;177(5):598-608. doi:10.7326/M23-3163 38648639

[26] Blevins C, Mullen L. Jane, John... Leslie? A Historical Method for Algorithmic Gender Prediction. Digital Humanities Quarterly; 2015:9.

[27] Unified Medical Language System. 2023. Accessed December 18, 2023. https://www.nlm.nih.gov/research/umls/index.html

[28] Betz-Stablein B, D’Alessandro B, Koh U, . Reproducible naevus counts using 3D total body photography and convolutional neural networks. Dermatology. 2022;238(1):4-11. doi:10.1159/000517218 34237739

[29] Santamaría L, Mihaljević H. Comparison and benchmark of name-to-gender inference services. PeerJ Comput Sci. 2018;4:e156. doi:10.7717/peerj-cs.156 33816809 PMC7924484

[30] Cinelli C, Hazlett C. Making sense of sensitivity: extending omitted variable bias. J R Stat Soc Series B Stat Methodol. 2020;82:39-67. doi:10.1111/rssb.12348

[31] Maxwell AE, Bastani R, Vida P, Warda US. Strategies to recruit and retain older Filipino-American immigrants for a cancer screening study. J Community Health. 2005;30(3):167-179. doi:10.1007/s10900-004-1956-0 15847243 PMC1810967

[32] Aroian KJ, Katz A, Kulwicki A. Recruiting and retaining Arab Muslim mothers and children for research. J Nurs Scholarsh. 2006;38(3):255-261. doi:10.1111/j.1547-5069.2006.00111.x 17044343 PMC1633727

[33] Alsan M, Garrick O, Graziani G. Does diversity matter for health? experimental evidence from Oakland. Am Econ Rev. 2019;109:4071-4111. doi:10.1257/aer.20181446

[34] Clark LT, Watkins L, Piña IL, . Increasing Diversity in Clinical Trials: Overcoming Critical Barriers. Curr Probl Cardiol. 2019;44(5):148-172. doi:10.1016/j.cpcardiol.2018.11.002 30545650

[35] Michos ED, Reddy TK, Gulati M, . Improving the enrollment of women and racially/ethnically diverse populations in cardiovascular clinical trials: an ASPC practice statement. Am J Prev Cardiol. 2021;8:100250. doi:10.1016/j.ajpc.2021.100250 34485967 PMC8408620

[36] Le D, Ozbeki H, Salazar S, Berl M, Turner MM, Price OA. Improving African American women’s engagement in clinical research: a systematic review of barriers to participation in clinical trials. J Natl Med Assoc. 2022;114(3):324-339. doi:10.1016/j.jnma.2022.02.004 35279325 PMC9189005

[37] Chatters R, Dimairo M, Cooper C, . Exploring the barriers to, and importance of, participant diversity in early-phase clinical trials: an interview-based qualitative study of professionals and patient and public representatives. BMJ Open. 2024;14(3):e075547. doi:10.1136/bmjopen-2023-075547 38508621 PMC10952868

[38] Ding EL, Powe NR, Manson JE, Sherber NS, Braunstein JB. Sex differences in perceived risks, distrust, and willingness to participate in clinical trials: a randomized study of cardiovascular prevention trials. Arch Intern Med. 2007;167(9):905-912. doi:10.1001/archinte.167.9.905 17502531

[39] Blehar MC, Spong C, Grady C, Goldkind SF, Sahin L, Clayton JA. Enrolling pregnant women: issues in clinical research. Womens Health Issues. 2013;23(1):e39-e45. doi:10.1016/j.whi.2012.10.003 23312713 PMC3547525

[40] Shields KE, Lyerly AD. Exclusion of pregnant women from industry-sponsored clinical trials. Obstet Gynecol. 2013;122(5):1077-1081. doi:10.1097/AOG.0b013e3182a9ca67 24104789

[41] Tobb K, Kocher M, Bullock-Palmer RP. Underrepresentation of women in cardiovascular trials: it is time to shatter this glass ceiling. Am Heart J Plus. 2022;13:100109. doi:10.1016/j.ahjo.2022.100109 38560055 PMC10978176

[42] Ellingrud K, Perez L, Petersen A, Sartori V. *Closing the Women’s Health Gap: A $1 Trillion Opportunity to Improve Lives and Economics.* McKinsey Health Institute; 2024.

[43] Inclusion of Women and Minorities as Participants in Research Involving Human Subjects. Accessed August 27, 2025.

[44] Sergeant A, Saha S, Shin S, . Variations in processes of care and outcomes for hospitalized general medicine patients treated by female vs male physicians. JAMA Health Forum. 2021;2(7):e211615. doi:10.1001/jamahealthforum.2021.1615 35977207 PMC8796959

[45] Ganguli I, Sheridan B, Gray J, Chernew M, Rosenthal MB, Neprash H. Physician Work Hours and the Gender Pay Gap - Evidence from Primary Care. N Engl J Med. 2020;383(14):1349-1357. doi:10.1056/NEJMsa2013804 32997909 PMC10854207

[46] NIH Policy and Guidelines on the Inclusion of Women and Minorities as Subjects in Clinical Research. Accessed September 25, 2024. https://grants.nih.gov/policy-and-compliance/policy-topics/inclusion/women-and-minorities/guideline#ii.-policy

[47] Anderson ML, Chiswell K, Peterson ED, Tasneem A, Topping J, Califf RM. Compliance with results reporting at ClinicalTrials.gov. N Engl J Med. 2015;372(11):1031-1039. doi:10.1056/NEJMsa1409364 25760355 PMC4508873

