# Supplementary material for: Gender Diversity of Research Teams and Clinical Trial Enrollment
Source: JAMA Netw Open. 2025 Oct 16;8(10):e2537667. doi: 10.1001/jamanetworkopen.2025.37667 (PMC12531878; doi:10.1001/jamanetworkopen.2025.37667)
Supplement: Supplement 1. — eFigure 1. Sample Construction eFigure 2. Time trends in women Principal Investigators and women participants eFigure 3. Enrollment of women participants in clinical trials according to gender composition of investigators in multi-Principal Investigator trials eFigure 4. Estimates of residual confounding bounds for select factors eFigure 5. Share of trials led by men versus women Principal Investigators that excluded pregnant patients, for drugs that are not contraindicated for pregnant women eTable 1. Comparison between included and excluded trials eTable 2. Findings using alternative thresholds for determining Principal Investigator gender eTable 3. Comparison of gender predictions using alternative algorithms eTable 4. Regression results using alternative algorithms to predict gender eTable 5. Estimate of residual confounding bounds eTable 6. Adjusted estimates with additional covariates eTable 7. Findings using additional methods of adjusting for disease eTable 8. Interactions with trial characteristics eMethods. Supplemental Methods eReferences [file jamanetwopen-e2537667-s001.pdf]

Supplemental Online Content

Gupta H, Jena AB, Sun EC. Gender diversity of research teams and clinical trial enrollment. *JAMA Netw Open*. 2025;8(10):e2537667. doi:10.1001/jamanetworkopen.2025.37667

eFigure 1. Sample Construction
eFigure 2. Time trends in women Principal Investigators and women participants
eFigure 3. Enrollment of women participants in clinical trials according to gender composition of investigators in multi-Principal Investigator trials
eFigure 4. Estimates of residual confounding bounds for select factors
eFigure 5. Share of trials led by men versus women Principal Investigators that excluded pregnant patients, for drugs that are not contraindicated for pregnant women
eTable 1. Comparison between included and excluded trials
eTable 2. Findings using alternative thresholds for determining Principal Investigator gender
eTable 3. Comparison of gender predictions using alternative algorithms
eTable 4. Regression results using alternative algorithms to predict gender
eTable 5. Estimate of residual confounding bounds
eTable 6. Adjusted estimates with additional covariates
eTable 7. Findings using additional methods of adjusting for disease
eTable 8. Interactions with trial characteristics
eMethods. Supplemental Methods
eReferences

This supplemental material has been provided by the authors to give readers additional information about their work.

© 2025 Gupta H et al. *JAMA Network Open.*

eTable 1: Comparison between included and excluded trials

	Trials Excluded form Analysis	Trials Included in Analysis	Difference 95% CI
Number of patients in trial, mean	664.8	665.6	0.8 [-21.4, 21.2]
Number of trial sites, mean	26.3	6.6	19.7 [18.7, 20.8]
Phase 3 or 4, %	42.2%	23.8%	18.4% [17.0%, 19.0%]
Amount of NIH funding, mean $ millions	4.0	3.4	2.1 [-4.6, 8.7]
Enrollment of women, %	49.0%	47.4%	0.8% [0.2%, 1.4%]

© 2025 Gupta H et al. *JAMA Network Open.*

eTable 2: Findings using alternative thresholds for determining Principal Investigator gender

	Adjusted share of participants that are women (95% CI)		
**Model**	**Trials led by women Principal Investigators**	**Trials led by men Principal Investigators**	**Absolute adjusted difference** (95% CI)
Primary model	54.1% (53.0% - 55.1%)	46.9% (46.3% - 47.5%)	7.3% (6.7% - 7.9%)
Model in which threshold for assigning Principal Investigator gender was > 90% probability	54.0% (52.9% - 55.1%)	46.7% (46.2% - 47.5%)	7.3% (6.8% - 7.9%)
Model in which threshold for assigning Principal Investigator gender was > 50% probability	53.8% (52.9% - 54.7%)	47.2% (46.6% - 47.8%)	6.8% (6.1% - 7.9%)

Notes: For our primary analysis, a Principal Investigator was considered to be a woman if the first name was associated with at least a 75% probability of being a woman and was considered to be a man if the first name was associated with at least a 75% probability of being a man. We performed sensitivity analyses in which the threshold for assigning a Principal Investigator was raised to 90% for a given gender (a stricter threshold for accuracy) and an additional analysis in which this threshold was lowered to 50% (a less strict threshold). Modifying this threshold did not affect the absolute adjusted difference in the share of participants that were women between trials led by women versus men Principal Investigators.

© 2025 Gupta H et al. *JAMA Network Open.*

eTable 3: Comparison of Gender Predictions Using Alternative Algorithms

	Agreement with primary name-to-gender algorithm (%)		
**Region**	*genderize.io*	*Gender API*	*NamSor*
All	99.3	98.9	98.8
Africa (n=412)	98.6	98.2	97.5
Americas (n=2,743)	99.8	99.4	99.3
Asia (n=3,621)	98.9	98.4	98.1
Europe (n=4,653)	99.4	99.0	99.2
Oceania (n=122)	99.1	100	99.1

Notes: Table reports the percent of trials where Principal Investigator gender, as predicted by our primary approach, matched gender predicted in each of three alternative algorithms (*genderize.io*, *Gender API*, and *NamSor* algorithms. The table shows rates of agreement for all the trials in our sample ("All") as well as for names predicted to come from five regions: Africa, Americas, Asia, Europe, and Oceania.). The region of a name is determined by a supplementary algorithm from *genderize.io*.

© 2025 Gupta H et al. *JAMA Network Open.*

eTable 4. Regression Results Using Alternative Algorithms to Predict Gender

	Percentage change in enrollment of women in trials with a woman Principal Investigator (standard error)			
	Primary algorithm	*genderize.io*	*NamSor*	*Gender API*
Full sample, unadjusted	0.0723*** (0.0057)	0.0721*** (0.0058)	0.0726*** (0.0057)	0.0720*** (0.0058)
Full sample, adjusted	0.0728*** (0.0031)	0.0723*** (0.0029)	0.0730*** (0.0029)	0.0724*** (0.0029)
European Names Only, unadjusted	0.0777*** (0.0091)	0.0786*** (0.0091)	0.0789*** (0.0092)	0.0768*** (0.0092)
European Names Only, adjusted	0.0786*** (0.0046)	0.0794*** (0.0042)	0.0798*** (0.0046)	0.0772*** (0.0042)
Non-European Names Only, unadjusted	0.0699*** (0.0075)	0.0691*** (0.0075)	0.0697*** (0.0075)	0.0701*** (0.0075)
European Names Only, adjusted	0.0702*** (0.0033)	0.0692*** (0.0031)	0.0701*** (0.0030)	0.0705*** (0.0033)

Notes: Table reports the estimated regression coefficients (and associated standard errors) from a linear regression in which the dependent variable is the share of women participants enrolled in a clinical trial. For the unadjusted analyses, the only independent variable was an indicator variable for whether the study Principal Investigator was predicted to be a woman, based on first name and using one of several name-to-gender algorithms. For the adjusted analyses, which are the primary results described in the study, the regression model included additional controls for study year and disease category. The Table shows that the estimated percentage change in enrollment of women in trials with a woman Principal Investigator did not vary according to the name-to-gender algorithm used or according to whether the name was European or non-European.

© 2025 Gupta H et al. *JAMA Network Open.*

eTable 5: Estimates of Residual Confounding Bounds

Treatment	Estimate, β	Standard error	t-value	$R^2_{Y\sim F}$	$RV_{q=1}$	$RV_{q=1,\alpha=0.05}$
Woman Principal Investigator	0.073	0.006	12.7	1.5%	11.6%	9.9%

Notes: $R^2_{Y\sim F}$ refers to the amount of variation in women's participation in trials explained by the gender of the Principal Investigator.

© 2025 Gupta H et al. *JAMA Network Open.*

eTable 6: Adjusted Estimates with Additional Covariates

Variable	Unadjusted Model	Primary Model	Primary Model with New Covariates
Woman Principal Investigator	0.0723*** (0.0057)	0.0728*** (0.0033)	0.0725*** (0.0033)
NIH Funding			0.0076 (0.0072)
Multicenter Trial			-0.0137* (0.0060)
Phase III/IV Trial			0.0268*** (0.0060)
Trial Size			$-5.18*10^{-8}$ $(4.51*10^{-8})$
Fixed Effects for Disease Being Studied	No	Yes	Yes
Fixed Effects for Trial Year	No	Yes	Yes

Notes: Table shows the estimated regression coefficients for three alternative models. For all three models, the outcome is the share of trial participants who are women, and the independent variable of interest is whether the trial Principal Investigator is a woman. The first column shows the result of an unadjusted analysis in which this is the only variable in the regression model. The second column shows the results of our primary analysis, which includes additional controls for the disease being studied and the year of trial enrollment. The third column presents the results when four additional variables are added to the regression model: whether the trial was a phase III/IV trial, whether the trial received funding from the National Institutes for Health (NIH), whether the trial was a multicenter trial, and the size of the trial. Standard errors are shown in parentheses. ***=p<0.001, **=p<0.01, *=p<0.05

© 2025 Gupta H et al. *JAMA Network Open.*

eTable 7: Findings using additional methods of adjusting for disease

	**Absolute adjusted difference between trials led by women versus men Principal Investigators** (95% CI)				
	Unadjusted	NIH Institute	Disease Category	Exclude sex-specific diseases	Primary Model
Estimate	7.2% [6.1%, 8.4%]	7.0% [6.0%; 7.9%]	7.1% [6.1%; 8.2%]	7.2% [1.8%; 12.5%]	7.3% [6.7%; 7.9%]
Method of adjusting for disease	No controls for disease	NIH Institute funding trial	One-digit ICD-9 code	Excluding sex-specific diseases	Three-digit ICD9-code

Notes: Table presents sensitivity analyses for how our baseline findings (primary model) were affected by additional methods for adjusting for disease. Table presents analyses in which adjustments were made for the NIH institute funding a trial, a proxy for diseases being studied; adjusting for disease category using 1-digit ICD-9 code, a coarse categorization of disease; adjusting for disease category using 3-digit ICD-9 code, a more granular categorization of disease that served as our primary model; and excluding diseases that are more specific to certain sexes (e.g., breast cancer, prostate cancer, ovarian cancer, etc.). The Table demonstrates that our observed finding of a higher share of women participants in trials led by women Principal Investigators is robust across these different methods of adjusting for diseases involved in a trial.

© 2025 Gupta H et al. *JAMA Network Open.*

eTable 8: Interactions with trial characteristics

	Interaction coefficient (95% CI)	
	Early stage trial (versus late stage)	Single site trial (versus multicenter)
Interaction between trial characteristic and indicator variable for whether a trial was led by a woman Principal Investigator	-0.7%<br>[-2.2%, 0.8%]	0.7%<br>[-1.0%, 2.4%]

Notes: Table presents findings from an analysis of whether the observed relationship between Principal Investigator gender and the proportion of trial participants that are women is modified by whether a trial is early versus late stage or single site versus multicenter, an effect modification or interaction analysis. The interaction coefficient between these trial characteristic variables and Principal Investigator gender was estimated from a regression model adjusting for year and disease fixed effects. Table demonstrates no statistically significant effect modification by these variables.

© 2025 Gupta H et al. *JAMA Network Open.*

eFigure 1: Sample Construction

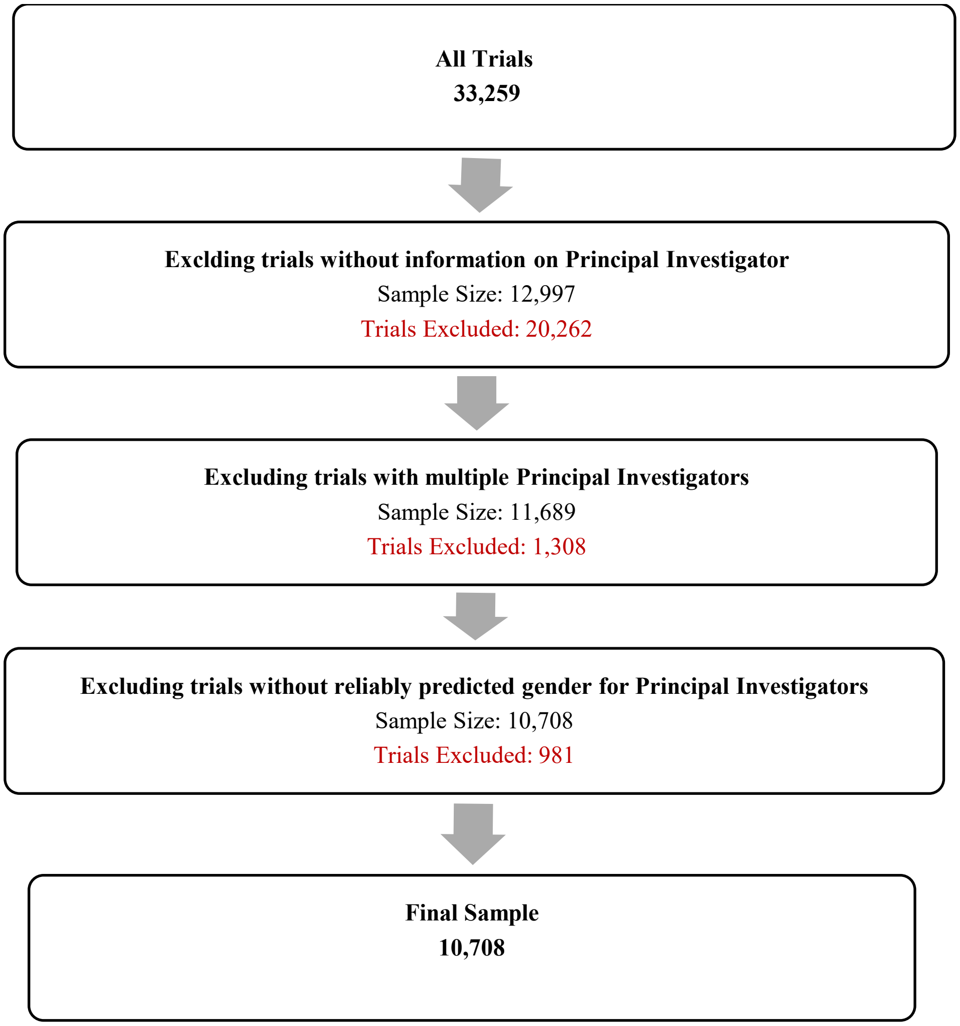

© 2025 Gupta H et al. *JAMA Network Open.*

eFigure 2: Time Trends in Women Principal Investigators and Women Participants

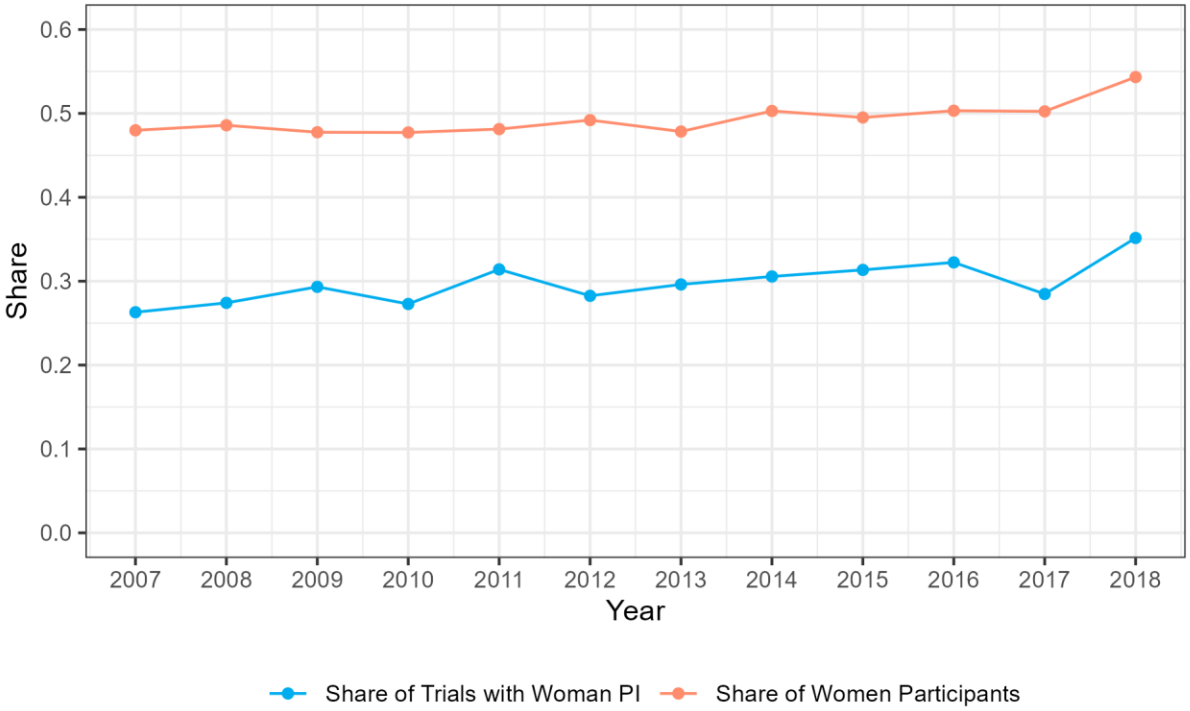

© 2025 Gupta H et al. *JAMA Network Open.*

eFigure 3: Enrollment of women participants in clinical trials according to gender composition of investigators in multi-Principal Investigator trials

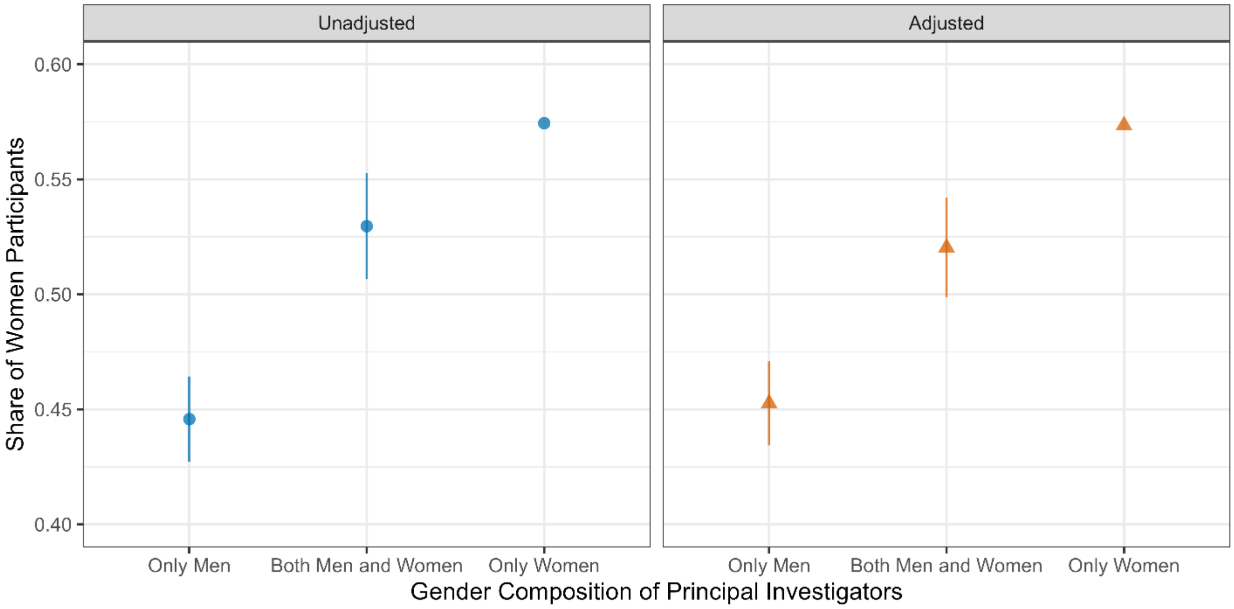

Notes: Figure shows the results of a sensitivity analysis performed on trials with more than one Principal Investigator (n=1,308). Figure presents the unadjusted and adjusted share of women participants for trials led by only men Principal Investigators, both men and women Principal Investigators, and trials with only women Principal Investigators. Bars represent 95% confidence intervals.

eFigure 4: Estimate of Residual Confounding Bounds for Select Factors

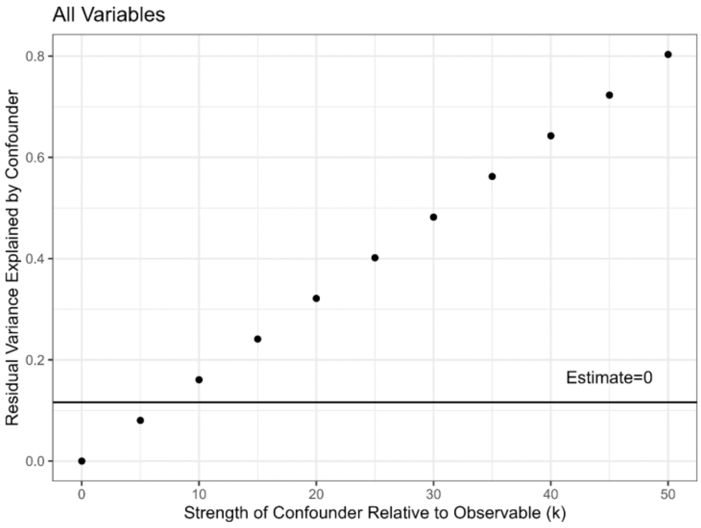

© 2025 Gupta H et al. *JAMA Network Open.*

eFigure 5: Share of trials led by men versus women Principal Investigators that excluded pregnant patients, for drugs that are not contraindicated for pregnant patients

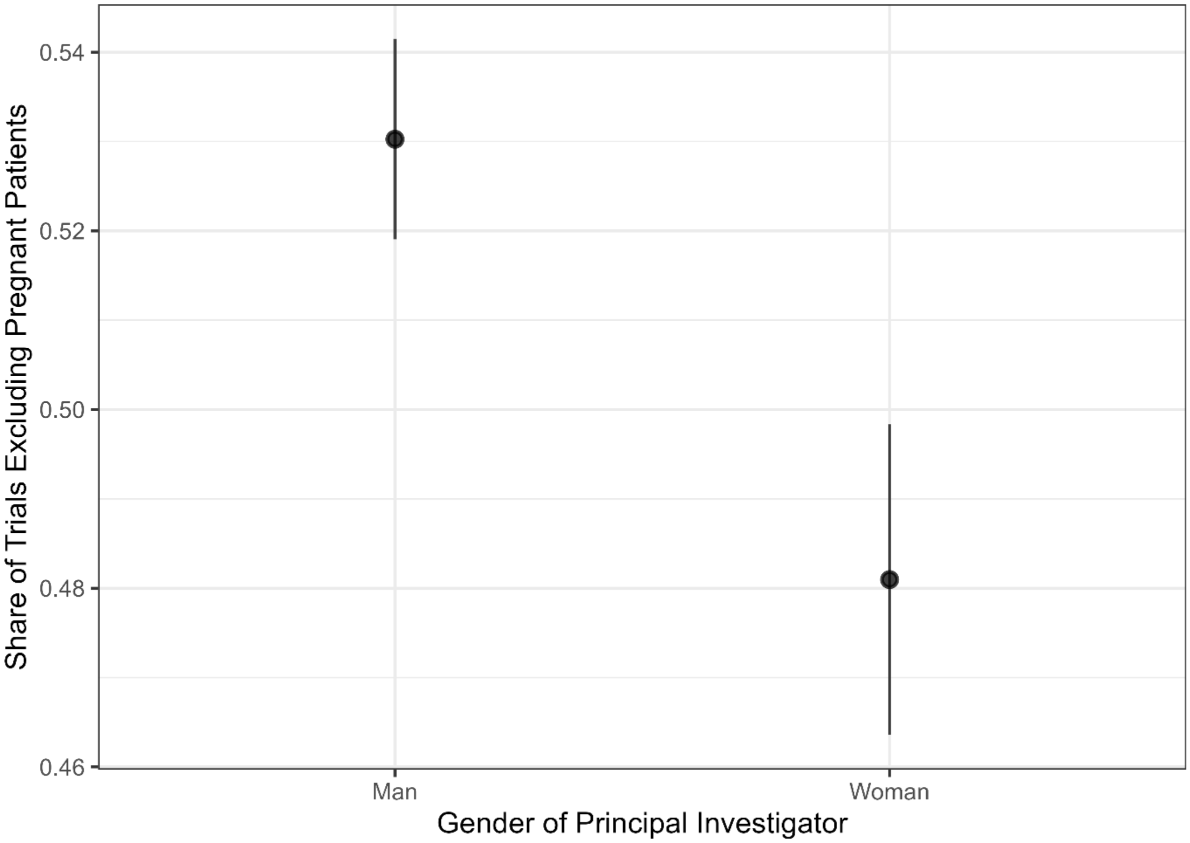

Notes: Figure presents the adjusted share of trials excluding pregnant patients for trials led by men versus women Principal Investigators (PIs). Bars represent 95% confidence intervals. The figure excluded trials for drugs that are contraindicated for pregnant women.

© 2025 Gupta H et al. *JAMA Network Open.*

eMethods

Identifying disease categories for trials

We examined the association between Principal Investigator gender and the share of women participants enrolled in a clinical trial, adjusting for variables that might confound this relationship. An important factor is the underlying disease(s) addressed by a given trial since diseases that primarily affect women, for example, would be expected to have a higher proportion of women participants. If Principal Investigator gender is not randomly allocated across disease areas (e.g., if Principal Investigators that are men are more likely to lead trials that focus on diseases preferentially affecting men), the failure to adjust for disease could bias our findings. We therefore identified the disease addressed by each trial in the AACT database by using the Unified Medical Language System from the National Library of Medicine to map MeSH (Medical Subject Heading) terms provided for each trial into International Classification of Diseases, Ninth Edition (ICD-9) codes.

MeSH terms are a controlled and hierarchically organized vocabulary produced by the National Library of Medicine used for indexing, cataloging, and searching of biomedical and health-related information. The AACT database provides a set of MeSH terms associated with each clinical trial. We extracted the MeSH major topic – the main topic of the trial as determined by the National Library of Medicine – for each trial. We then mapped MeSH terms to ICD-9 administrative diagnosis codes (a nomenclature for classifying diseases) using the Unified Medical Language System, a large biomedical thesaurus linking terms in one medical vocabulary to terms in another medical vocabulary that have the same meaning or refer to the same concept. For each trial, we queried the Unified Medical Language System with MeSH terms and obtained the associated ICD-9 codes with that MeSH term.

© 2025 Gupta H et al. *JAMA Network Open.*

Use of Alternative Algorithms to Predict Gender

In our primary analysis, we used the *gender*[1] package in R, which uses large historical datasets to predict gender based on first names. These include sources such as the Social Security Administration, IPUMS-USA, and the North Atlantic Population Project. Although this tool offers a comprehensive method for evaluating gender, it may have two limitations. First, the underlying data is almost exclusively based on the U.S., Northern Europe, and Western Europe, which could limit its applicability to first names with other origins. Second, this method fails to capture recent advances in gender prediction methodology.

To examine the robustness of our findings, we replicated our methodology and baseline analysis using three other name-to-gender algorithms: *Gender API, genderize.io, and Namsor*. We selected these tools based on the recommendation of recent studies that evaluate a larger suite of gender prediction algorithms.[2,3] In addition, these algorithms are commonly used in both academia and industry. Moreover, *Gender API* and *Namsor* were explicitly designed to cover a large set of countries and are based on more modern datasets, and thus should not have some of the limitations present in the *gender* package used for the primary analysis.

Gender API provides an API that predicts gender based on first name. The prediction is based on an array of publicly available and governmental data from 191 countries, including manual additions/corrections. Specifically, the dataset is reported to contain 6,084,389 validated names from 191 different countries gathered from about 10,000,000 datasets per month to improve the quality of gender prediction. (https://gender-api.com/en/).

NamSor is a similar algorithm that classifies gender based on name and can recognize the linguistic or cultural origin of names. NamSor (https://namsor.app/) covers all languages,

© 2025 Gupta H et al. *JAMA Network Open.*

alphabets, countries, and regions. The underlying data consists of 1.3 million unique given names extracted from baby name statistics in each country, plus sociolinguistic information (morphology, language, ethnicity, etc.) to extract semantics, which allows it to predict gender for complex cases. The algorithm uses both standard statistical prediction methods as well as artificial intelligence to classify gender. The algorithm has also been used extensively in academic research.[4] Finally, *genderize.io* ((https://genderize.io/) is a name-to-gender algorithm that predicts gender based on 908,290,909 names from multiple countries across all continents. This algorithm has also been used extensively for research.[5,6]

We first compared the predictions of our primary name-to-gender algorithm to the predictions of these alternative algorithms for the Principal Investigator names used in our study. **eTable 3** shows the extent to which the gender predicted in our baseline approach (using the *gender* package in R) matched the predicted gender from these alternative algorithms. In addition, *genderize.io* predicts the region of a given name; therefore, we presented rates of agreement between algorithms, by region. This directly assessed the suitability of our primary name-to-gender algorithm to disambiguate gender for non-European names. **eTable 3** demonstrates a high degree of agreement—approximately 99%—between our primary approach and the alternative algorithms. Moreover, the table also shows that this high degree of agreement is not confined to European names and indeed extends to all of the regions that we examine.

We then investigated the sensitivity of our primary findings – the percentage change in enrollment of women in trials led by a woman Principal Investigator – to the choice of gender prediction algorithm. To do so, we re-estimated our main regression model using the predicted gender of Principal Investigators from each of the alternative algorithms. The results are shown in **eTable 4**. The table also presents results stratifying our analyses by European and non-

© 2025 Gupta H et al. *JAMA Network Open.*

European names, using the region predicted by the *genderize.io* algorithm. The table shows that our results are invariant both to the choice of algorithm used as well as to subgroup analyses focused on either European or non-European names.

Analysis of Sensitivity to Unobserved Confounding

Omitted variable bias, confounding due to unobserved features, is a potential source of bias in observational studies. For example, if clinical trials led by women Principal Investigators differ systematically in other factors (e.g., the geographical location of trials) that are also correlated with the share of trial participants that are women (the outcome), the estimated association of having a woman as a Principal Investigator would be biased. One way to address the potential importance of unmeasured confounders is to estimate the explanatory power of unobserved confounders that would be necessary to explain away a given study's results; researchers can then assess the extent to which a given study is sensitive to confounding. We examined the sensitivity of our primary estimates to different assumptions about unobserved confounders utilizing the method proposed by Cinelli and Hazlett.[7]

As a brief exposition, our study examines the association between the share of women participants in a clinical trial (Y) and whether the trial has a woman Principal Investigator (F), after adjusting for (i.e., conditional on) other variables such as disease and year fixed effects (a vector, X). Let β denote our estimate of the association between Y and F, conditional on X.[1] The key concern is that there is some unobserved confounder Z that influences both Y and F, thereby biasing β due to omitted variable bias. The extent of this bias depends on two objects:

[1] For ease of exposition, we do not use the conditional on X notation where appropriate, but both our exposition and analyses are conditional on X.

© 2025 Gupta H et al. *JAMA Network Open.*

1. The extent to which Z explains the variation in the gender of the Principal Investigator: $R^2_{F\sim Z}$
2. The extent to which Z explains the variation in the share of women participants, conditional on the gender of the Principal Investigator: $R^2_{Y\sim Z|F}$

Of course, the main challenge is that we do not observe Z and so cannot precisely calculate these two objects. Instead, we can analyze how our estimates would change for different values of $R^2_{F\sim Z}$ and $R^2_{Y\sim Z|F}$.

Following the recommendations in Cinelli and Hazlett (2019), we start by examining the Robustness Value (RV). Assuming that a confounder has an equal association with the gender of a Principal Investigator and share of women participations, $R^2_{F\sim Z} = R^2_{Y\sim Z|F}$, the RV measures how strong of an association of Z with F (and, equivalently, Y) is required to reduce the estimated effect by 100*q%. When q=1, then this corresponds to the case where the true value of β is 0. The results of our confounding analysis are presented in **eTable 5** below.

We estimate two Robustness Values, $RV_{q=1}$ and $RV_{q=1,\alpha=0.05}$. The former is the degree of confounding required to completely explain away our results (i.e., to make β = 0), while the latter is the degree of confounding required to make our results statistically insignificant (i.e., to make β statistically insignificant from 0 at the 5% level of significance). $RV_{q=1}$ equals 11.6%, meaning that an unmeasured confounder would need to be strong enough to explain 11.6% of the residual variation in *both* the gender of the Principal Investigator and share of women participants in order to completely explain away the estimated relationship between women Principal Investigators and share of women participants. Similarly, $RV_{q=1,\alpha=0.05}$ equals 9.9%,

© 2025 Gupta H et al. *JAMA Network Open.*

meaning that an unmeasured confounder would need to be strong enough to explain 9.9% of this residual variation in order for our estimate to lose statistical significance.

To evaluate whether such strong confounders are plausible in our setting, we estimate the residual variation in the gender of Principal Investigators and the share of women participants explained by observable features that we did not include in our primary analysis (denoted by *W*). We can then assess the plausibility of strong unobserved confounders relative to these newly included observed confounders. For example, we can state that unobserved confounders would need to explain k times the variation explained by one or all of these observable features. These concepts are formally defined as in Cinelli and Hazlett (2019) as:

$$k_F = \frac{R^2_{F\sim Z}}{R^2_{F\sim W}}$$

$$k_Y = \frac{R^2_{Y\sim Z|F}}{R^2_{Y\sim W|F}}$$

where k_F is how much of the variation in the gender of the Principal Investigator is explained by Z relative to that explained by observable feature(s) W. Similarly, k_Y is the amount of variation in the share of women participants explained by Z relative to that explained by W. We can directly measure $R^2_{F\sim W}$ and $R^2_{Y\sim W|F}$ by including W in our regression model. Then, for different assumptions about k_F and k_Y, we can obtain $R^2_{F\sim Z}$ and $R^2_{Y\sim Z|F}$, allowing us to assess the importance of any unobserved confounding by comparing these objects to RV defined and estimated above.

To implement this approach, we selected four additional variables (*W*) not included in our original model: whether a trial was a phase III/IV trial, whether the trial was funded by the National Institutes of Health, trial size (number of patients enrolled), and whether the trial was a multicenter trial.

© 2025 Gupta H et al. *JAMA Network Open.*

As a first step, we re-estimated our primary regression model with the addition of these variables, in order to see if their inclusion would change our results. On the whole, we find that the inclusion of these variables did not significantly alter our findings (**eTable 5**), despite the fact that each of them may be expected to have an impact on clinical trial enrollment of women. For instance, until 1993, women were excluded from early-stage trials due to a FDA guidance, which would suggest clinical trial phase would be an important predictor of trial enrollment of women. Similarly, over the years the NIH has implemented several policies aimed at improving representation of women for the studies it funds, suggesting that the presence of NIH funding may have an impact of trial enrollment of women..[8] The robustness of our results to the inclusion of observed variables expected to be strongly associated with the outcome suggests informally that our results are robust to potential unobserved variables with the same explanatory power as these observed variables.

More formally, in **eFigure 4** below, we report the $max\left(R^2_{F\sim Z}, R^2_{Y\sim Z|F}\right)$ for the entire group of variables W (the four additional variables we added to the primary analysis: NIH funding, phase III/IV trial, trial size, and multicenter trial) and different values of $k = k_F = k_Y$. Note that by considering the maximum of these variables, we are imposing a stricter requirement on ourselves: a strong association with *either* F or Y (instead of both) will suggest strong bias in our test. If we find that $max\left(R^2_{F\sim Z}, R^2_{Y\sim Z|F}\right) < RV$ presented in the table above, then it implies that our estimates would not be 0 even if there exists confounders that explain k times more the variation in both the gender of the Principal Investigator and share of women participants than explained by W. Overall, we find that any unobserved confounding must be at least 10 times more explanatory than the four additional variables *combined* to explain our results. Thus, our results our robust to unobserved confounding up to a very high threshold.

© 2025 Gupta H et al. *JAMA Network Open.*

© 2025 Gupta H et al. *JAMA Network Open.*

eReferences

1. Blevins C, Mullen L. Jane, John... Leslie? A Historical Method for Algorithmic Gender Prediction. *Digital Humanities Quarterly.* 2015;9(3).
2. Betz-Stablein B, D'Alessandro B, Koh U, et al. Reproducible Naevus Counts Using 3D Total Body Photography and Convolutional Neural Networks. *Dermatology.* 2022;238(1):4-11.
3. Santamaria L, Mihaljevic H. Comparison and benchmark of name-to-gender inference services. *PeerJ Comput Sci.* 2018;4:e156.
4. Bursztyn L, Chaney T, Hassan TA, Rao A. The Immigrant Next Door. *American Economic Review.* 2024;114(2):348-384.
5. Waldhorn I, Bomze D, Ben-Aharon I, et al. Gender Gap in Leadership of Clinical Trials. *JAMA Intern Med.* 2023;183(12):1406-1408.
6. Muquith M, Pham T, Espinoza M, Hsiehchen D. Representation of Investigators by Gender Among Authors of Phase 3 Oncology Trials Worldwide. *JAMA Netw Open.* 2022;5(2):e220031.
7. Cinelli C, Hazlett C. Making Sense of Sensitivity: Extending Omitted Variable Bias. *Journal of the Royal Statistical Society Series B: Statistical Methodology.* 2020;82(1):39-67.
8. NIH Policy and Guidelines on The Inclusion of Women and Minorities as Subjects in Clinical Research. https://grants.nih.gov/policy-and-compliance/policy-topics/inclusion/women-and-minorities/guideline#ii.-policy. Accessed September 25, 2024.

© 2025 Gupta H et al. *JAMA Network Open.*
