# Supplementary material for: Gender Diversity of Research Teams and Clinical Trial Enrollment
Source: JAMA Netw Open. 2025 Oct 16;8(10):e2537667. doi: 10.1001/jamanetworkopen.2025.37667 (PMC12531878; doi:10.1001/jamanetworkopen.2025.37667)
Supplement: Supplement 2. — Data Sharing Statement [file jamanetwopen-e2537667-s002.pdf]

Data Sharing Statement

Gupta. Gender Diversity of Research Teams and Clinical Trial Enrollment. *JAMA Netw Open*. Published October 16, 2025. doi:10.1001/jamanetworkopen.2025.37667

Data
Data available: No
